# Correction: Covalent organic functionalization of graphene nanosheets and reduced graphene oxide *via* 1,3-dipolar cycloaddition of azomethine ylide

**DOI:** 10.1039/d1na90092g

**Published:** 2021-10-06

**Authors:** Luca Basta, Aldo Moscardini, Filippo Fabbri, Luca Bellucci, Valentina Tozzini, Silvia Rubini, Andrea Griesi, Mauro Gemmi, Stefan Heun, Stefano Veronesi

**Affiliations:** NEST, Istituto Nanoscienze-CNR, Scuola Normale Superiore Piazza S. Silvestro 12 56127 Pisa Italy luca.basta1@sns.it stefano.veronesi@nano.cnr.it +39 050 509882; Istituto Officina dei Materiali CNR, Laboratorio TASC Area Science Park – S S 14, km 163.5 I-34012 Trieste Italy; Department of Chemistry, Life Sciences and Environmental Sustainability, University of Parma Parco Area delle Scienze 17/A 43124 Parma Italy; Center for Nanotechnology Innovation@NEST, Istituto Italiano di Tecnologia Piazza S. Silvestro 12 56127 Pisa Italy

## Abstract

Correction for ‘Covalent organic functionalization of graphene nanosheets and reduced graphene oxide *via* 1,3-dipolar cycloaddition of azomethine ylide’ by Luca Basta *et al.*, *Nanoscale Adv.*, 2021, DOI: 10.1039/D1NA00335F.

The authors regret the omission of a funding acknowledgement in the original article. This acknowledgement is given below.

“This research was funded by EU-H2020 FETPROACT LESGO (Agreement No. 952068), and by the Italian Ministry of University and Research (project MONSTRE-2D PRIN2017 KFMJ8E)”

The Royal Society of Chemistry apologises for these errors and any consequent inconvenience to authors and readers.

## Supplementary Material

