# Supplementary material for: Covalent organic functionalization of graphene nanosheets and reduced graphene oxide via 1,3-dipolar cycloaddition of azomethine ylide
Source: Nanoscale Adv. 2021 Aug 30;3(20):5841–52. doi: 10.1039/d1na00335f (PMC9418116; doi:10.1039/d1na00335f)
Supplement: NA-003-D1NA00335F-s001 [file NA-003-D1NA00335F-s001.pdf]

Electronic Supplementary Material (ESI) for Nanoscale Advances.
This journal is © The Royal Society of Chemistry 2021

Covalent Organic Functionalization of Graphene Nanosheets and Reduced Graphene Oxide via 1,3-Dipolar Cycloaddition of Azomethine Ylide

Electronic Supplementary Information

Luca Basta, Aldo Moscardini, Filippo Fabbri, Luca Bellucci, Valentina Tozzini, Silvia Rubini, Andrea Griesi, Mauro Gemmi, Stefan Heun, Stefano Veronesi†*
(*: luca.basta1@sns.it; †: stefano.veronesi@nano.cnr.it)

Dispersion of GNS

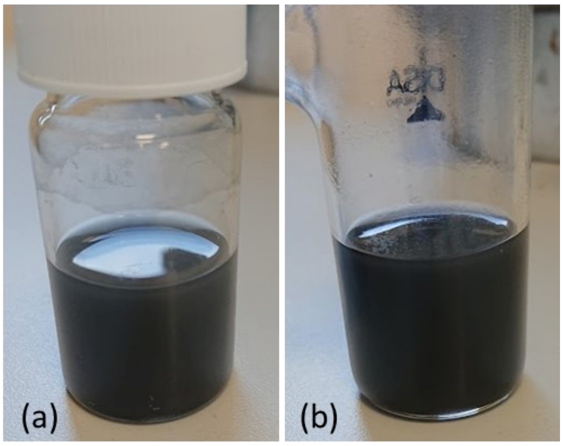

Figure S1. Dispersion of GNS in NMP after (a) sonication and (b) homogenization.

DLS Measurement

In order to compare 1-methyl-2-pyrrolidinone (NMP) and N,N-dimethylformamide (DMF) as dispersion solvents, as well as sonication and homogenization as dispersion techniques, we performed dynamic light scattering (DLS) measurements on dispersed graphene nanosheets (GNS) (0.25 mg mL^{-1}). A commercially available Malvern Zetasizer Nano ZS equipped with a 633 nm HeNe laser was used for this purpose. In the automatic setting, the system optimizes the focal position and the attenuation of the incident beam before the data acquisition. The Zetasizer software uses a set of algorithms to analyze the correlation of scattering events and gives as output the relative intensity of light scattered by particles of a given average lateral dimension. The values are collected in Table S1. A better dispersion was achieved in NMP with respect to DMF, because of its optimal surface tension. Among the two dispersion techniques, homogenization results in a faster method with respect to sonication, allowing to obtain almost the same result in half the time (comparing the results after 30 and 60 minutes).

Solvent	*Dispersion technique*	*Start*	*After 30 min*	*After 60 min*	*After 120 min*
**NMP**	*sonication*	(850 ± 100) nm	-	(600 ± 100) nm	(500 ± 100) nm
	homogenization	(850 ± 50) nm	(600 ± 40) nm	(400 ± 20) nm	-
**DMF**	*sonication*	(950 ± 150) nm	-	(850 ± 150) nm	(800 ± 150) nm
	homogenization	(950 ± 150) nm	(800 ± 50) nm	(600 ± 50) nm	-

Table S1. Average lateral dimension of GNS dispersed in NMP or DMF, via sonication (60 W) or homogenization (30000 rpm). (-) : not measured.

EDX Measurements

Energy-dispersive X-ray spectroscopy (EDX) spectra were acquired after washing the samples with ethanol, using carbon film-supported copper grids. Therefore, signal from the C and Cu of the grid is expected, and thus a quantitative analysis is not possible. The detection of the N and O peaks from functionalized graphene confirms the presence of azomethine ylides on graphene's surface, which present functional groups containing nitrogen and oxygen.

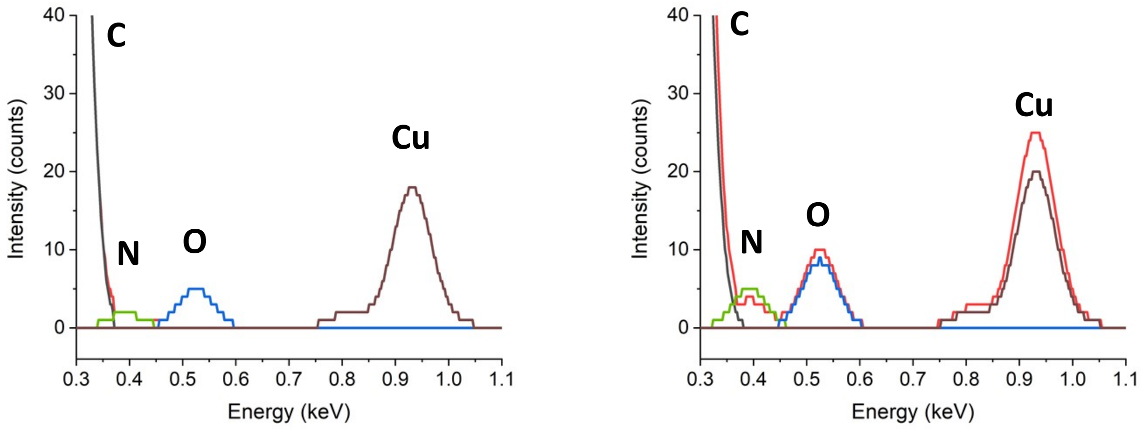

(a) (b)

Figure S2. EDX spectra (red line) of functionalized (a) GNS in NMP and (b) GNS in DMF (colored lines: deconvolution). Only a zoom of the region of interest is shown.

EELS Measurements

The electron energy loss spectroscopy (EELS) spectra of the N-K edge of functionalized GNS and rGO show two bands, centered at 402 eV and at 410 eV, assigned respectively to π* and σ* contributions. Like in the EDX measurements, signal from nitrogen is expected only from the azomethine ylides, and its presence confirms the success of the functionalization.

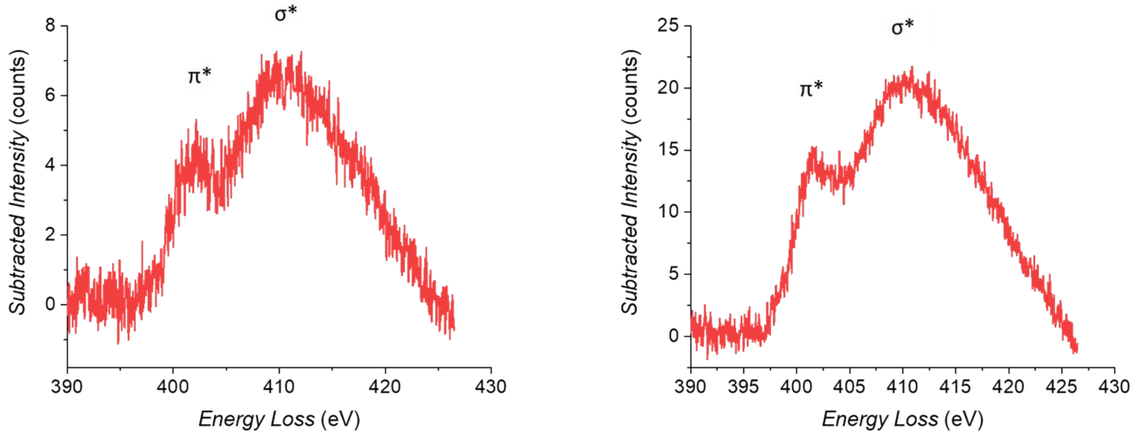

(a) (b)

Figure S3. EELS spectra of functionalized (a) GNS in DMF and (b) rGO in DMF. The background was subtracted from the spectra in order to highlight the K-edges of N.

Raman Measurements

In order to properly assign the Raman features that arise from the functionalization by 1,3-DC of azomethine ylide, Raman spectroscopy was performed also on residuals of NMP and DMF. Both solvents were dropcasted on clean substrates, which were then dried under vacuum, and analyzed. Noticeably, the intensities of the spectra are evidently small, with respect to the intensities of the spectra of pristine and functionalized graphene. No characteristic peaks are present in the intermediate zone of the spectrum, where peaks 1 - 4 were detected on functionalized GNS and rGO. This result confirms that this set of new peaks originates from the azomethine yilde, and not from the solvents.

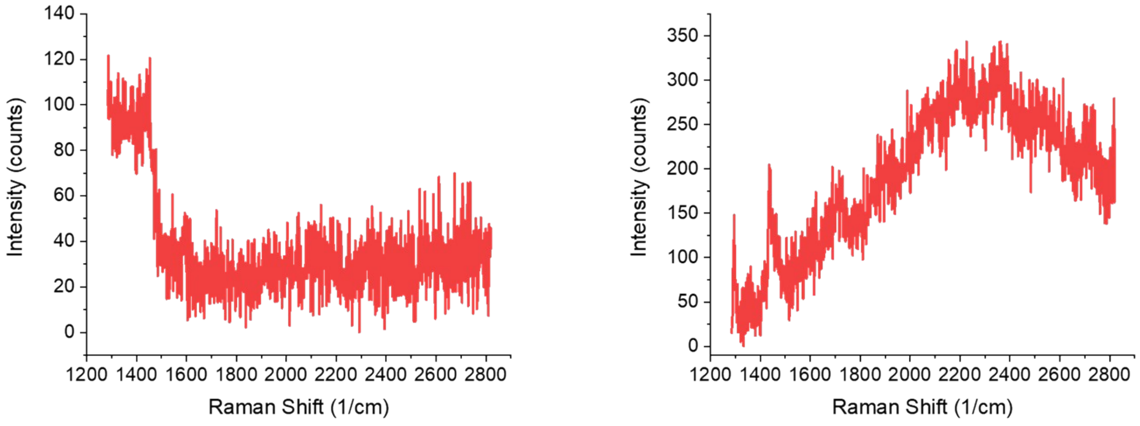

(a) (b)

Figure S4. Raman spectra of residuals of (a) NMP and (b) DMF after drying under vacuum.

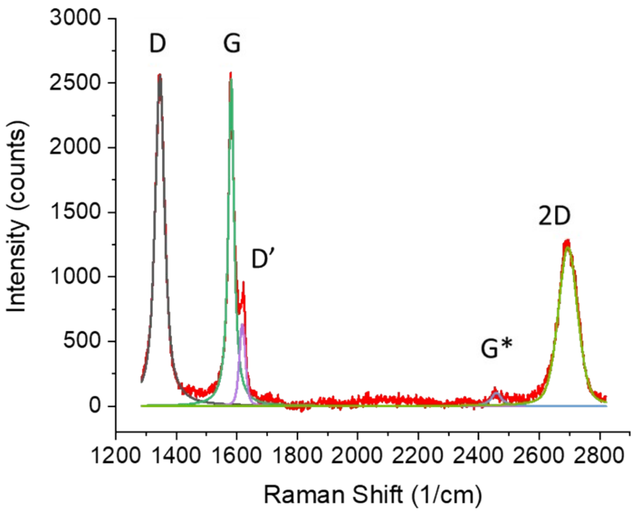

Figure S5. Raman spectrum of pristine GNS dispersed in DMF, and the fit showing the five characteristic peaks of graphene (each peak is labeled).

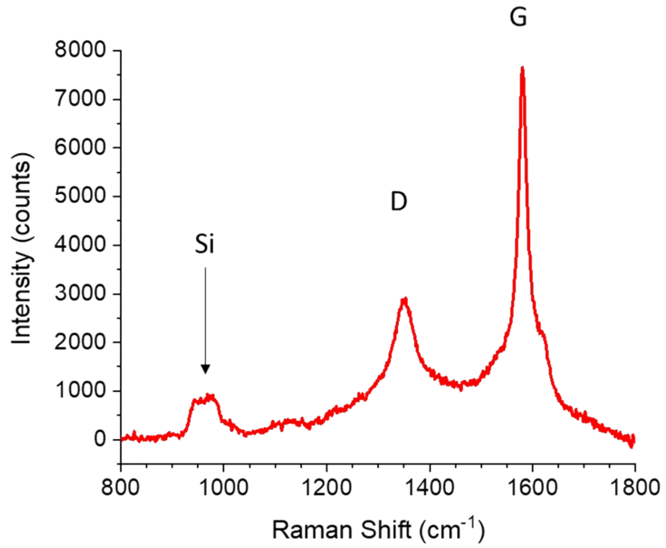

Figure S6. Raman spectrum of functionalized GNS in DMF. The shoulder [920 – 1050] cm^{-1} coming from the Si signal of the substrate is indicated by an arrow.

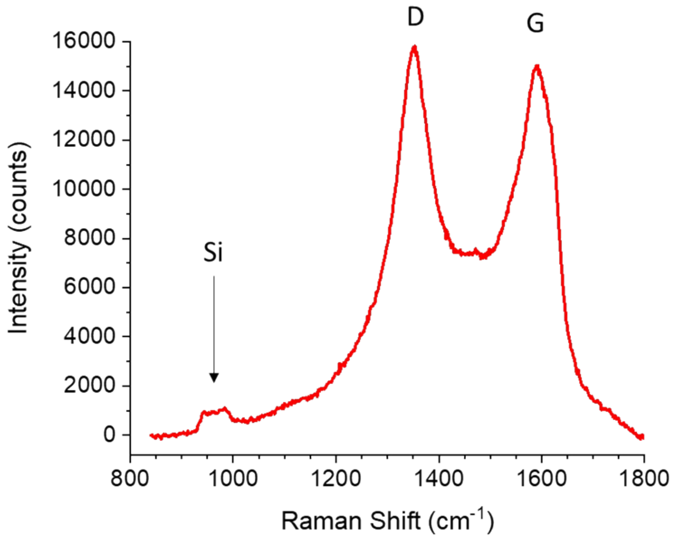

Figure S7. Raman spectrum of functionalized rGO. The shoulder [920 – 1050] cm^{-1} coming from the Si signal of the substrate is indicated by an arrow.

XPS Measurements

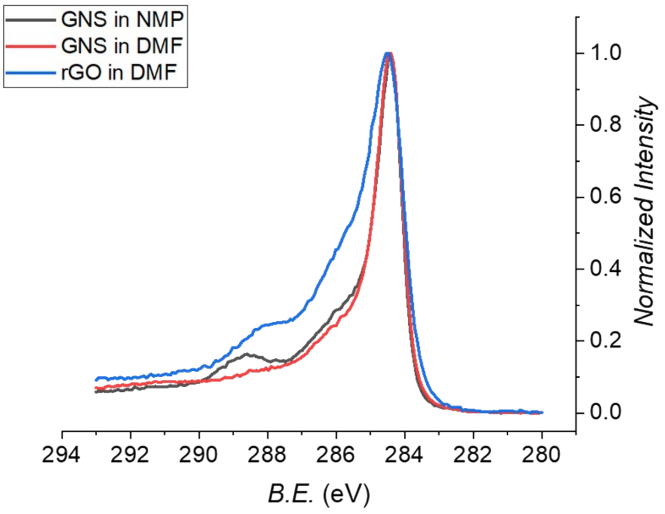

Figure S8. XPS spectra of the C 1s core level from functionalized GNS in NMP, GNS in DMF, and rGO in DMF. The data was normalized for better comparison of the spectra.

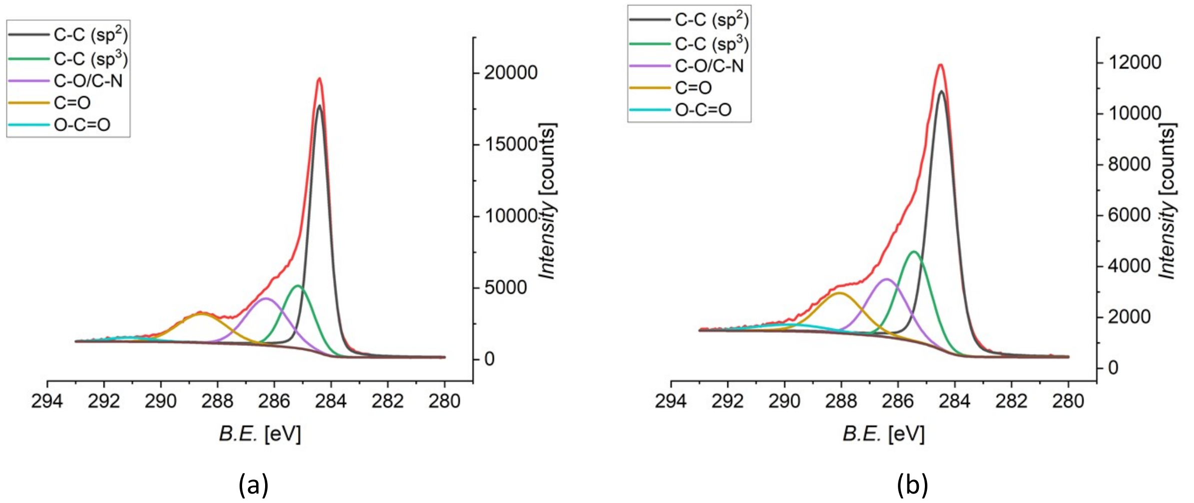

Figure S9. XPS spectra of the C 1s core level from functionalized (a) GNS in NMP and (b) rGO in DMF (Shirley-type background in brown).

The X-ray photoemission spectroscopy (XPS) C 1s spectrum taken on functionalized rGO shows a higher intensity of the component of C-C sp^3, consistent with a higher degree of functionalization and a higher presence of defects in the initial structure of rGO.

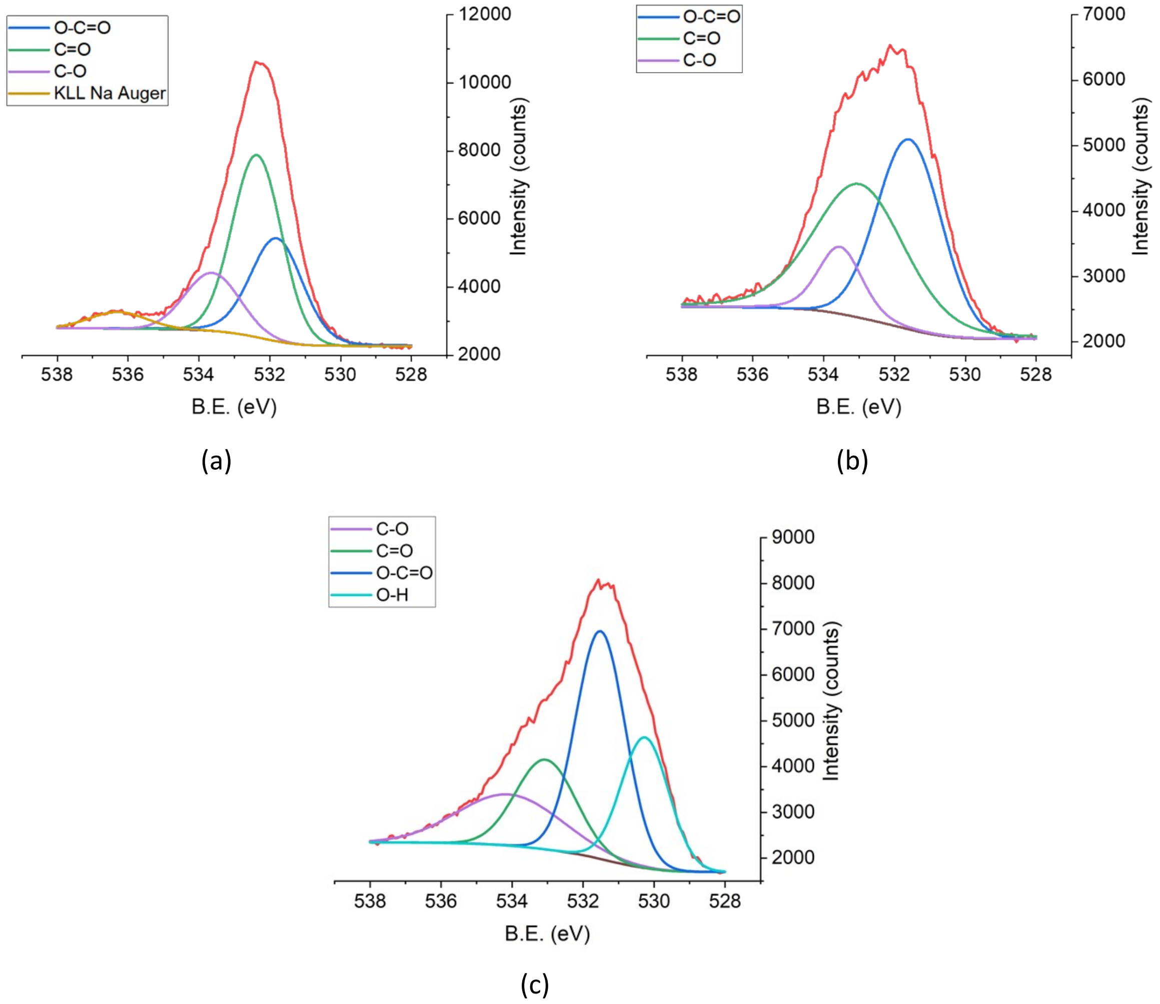

Figure S10. XPS spectra of the O 1s core level from functionalized (a) GNS in NMP, (b) GNS in DMF, and (c) rGO in DMF. The fit of each spectrum is shown, as well (Shirley-type background in brown).

From the O 1s spectrum taken on functionalized GNS in NMP, the overlapping of the KLL Na Auger can be distinguished (Na is used as base in the reaction procedure of the 1,3-DC). From the spectrum collected on functionalized rGO, the peak coming from the O-H bonding is noticeable, as a result of the higher abundance of this functional group already in the initial structure of rGO.

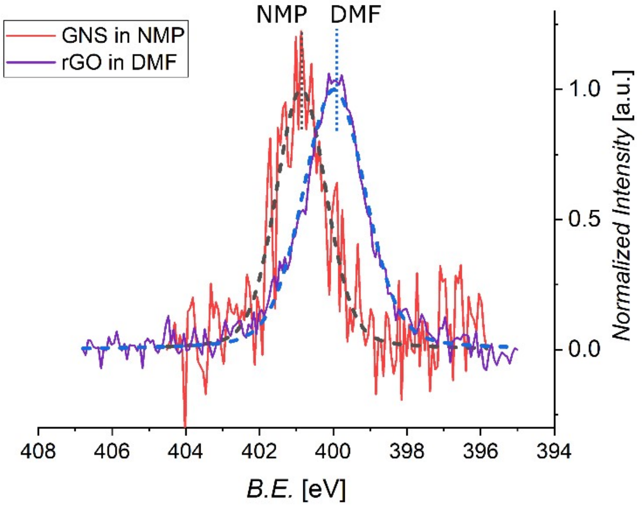

Figure S11. XPS spectra of the N 1s core level from pristine GNS in NMP and pristine rGO in DMF. The fit of each spectrum is shown, as well.

In order to measure the B.E. of the solvents, pristine GNS dispersed in NMP and pristine rGO dispersed in DMF were deposited *via* dropcasting onto silicon substrates, following the same procedure used for functionalized samples, and then analyzed with XPS. From the fitting of the N 1s core level, the B.E. of both NMP and DMF were calculated, resulting in 400.9 eV for NMP and 400.0 eV for DMF. These values were then used as references in the fitting of functionalized samples.

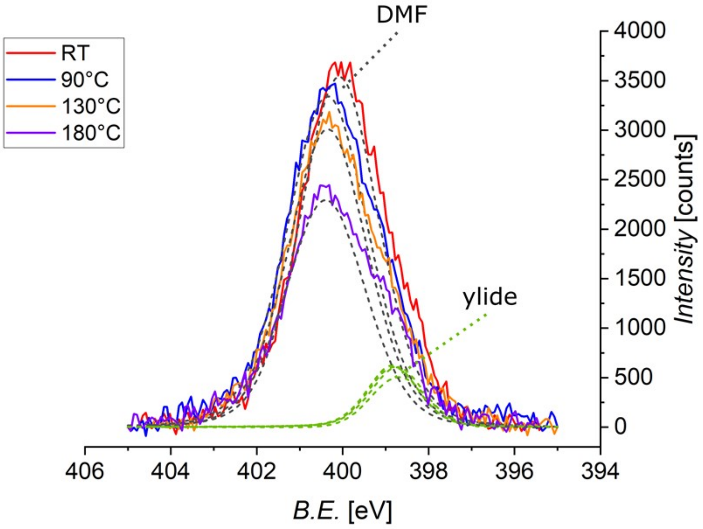

Figure S12. XPS spectra of the N 1s core level from functionalized GNS in DMF at room temperature, and after annealing at 90 °C, 130 °C, and 180 °C. The fit of each spectrum is shown, as well (peak from DMF in black dash line, peak from the ylide in green dash line).

Comparing the N 1s core level acquired after annealing of functionalized GNS in DMF, the decrease of the intensity of the peak centered around 400.1 eV (black dash line in Figure S11) indicates the desorption of the solvent, while the stability of the functionalization is shown by a much more constant intensity of the peak centered at 398.8 eV (green dash line in Figure S11).
